# Analysis of the talocrural and subtalar joint motions in patients with medial tibial stress syndrome

**DOI:** 10.1186/s13047-015-0084-7

**Published:** 2015-07-01

**Authors:** Kei Akiyama, Byungjoo Noh, Mako Fukano, Shumpei Miyakawa, Norikazu Hirose, Toru Fukubayashi

**Affiliations:** Graduate School of Sport Sciences, Waseda University, 2-579-15 Mikajima, Tokorozawa, Saitama 359-1192 Japan; Japan Institute of Sports Sciences, Sports Science, 3-15-1, Nishigaoka, Kita-ku 115-0056 Japan; Department of Sports Medicine, Graduate School of Comprehensive Human Sciences, University of Tsukuba, 1-1-1 Tennodai, Tsukuba, Ibaraki 305-8577 Japan; Faculty of Sport Sciences, Waseda University, 2-579-15 Mikajima, Tokorozawa, Saitama 359-1192 Japan

**Keywords:** Medial tibial stress syndrome, Subtalar joint, Talocrural joint, 3D to 2D registration technique

## Abstract

**Background:**

The rearfoot motion during sports activities in patients with the medial tibial stress syndrome (MTSS) is unknown. This study aimed to investigate the difference in kinematics of the rearfoot in MTSS patients (eight male soccer players) and control participants (eight male soccer players) during a forward step.

**Methods:**

Sixteen male soccer players, including eight players with MTSS, participated. Forward step trials were recorded with cineradiographic images obtained at a sampling rate of 60 Hz. Geometric bone models of the tibia and talus/calcaneus were created from computed tomography scans of the distal part of one lower limb. Following a combination of approaches, anatomical coordinate systems were embedded in each bone model. The talocrural joint motion (relative motion of the talus with respect to the tibia) and subtalar joint motion (relative motion of the calcaneus with respect to the talus) were examined.

**Results:**

A significantly larger range of internal/external rotation and inversion/eversion motion was observed in the subtalar joint of MTSS patients compared to healthy controls (*P* < 0.05) from heel contact to heel off. There were no significant differences between the MTSS patients and healthy participants in the ranges of all talocrural joint angles during the forward step.

**Conclusion:**

Our results indicate that the range of subtalar joint motion is greater in patients with MTSS during the stance phase of the forward step. The kinematic results obtained of this study may have important clinical implications and add quantitative data to an in vivo database of MTSS patients.

## Background

The medial tibial stress syndrome (MTSS), first described by Mubarak et al. in 1982 [1], is one of the most common painful exercise-induced leg injuries [2, 3]. MTSS encompasses several injuries, including medial tibial periostitis, soleus enthesopathy, and shin splints [3, 4]. However, the aetiology of this condition has been debated for years, and MTSS continues to be an intriguing and confusing pathological condition. Pain associated with MTSS commonly occurs in the middle or distal third of the posteromedial border of the tibia [3, 5]. The site of pain is typically spread over a minimum of 5 cm [6]. In contrast, a stress fracture has an area of focal tenderness of only 2–3 cm [7, 8]. Advanced MTSS patients commonly experience pain at the onset of activity [9]. Military studies have indicated that the incidence of MTSS is 6–38 % [5, 6, 10]. The recovery time for the resolution of MTSS ranges from 4 weeks to 18 months [9, 11], and MTSS is often refractory to conservative management. MTSS is proposed to be triggered by internal (tibial alignment, flat foot, low medial longitudinal arch, and forefoot varus) and external factors (over work, sports surface, shoes) [5, 6, 12], although these causes remain largely speculative. A previous study suggested the use of an evidence-based prevention method [13], but effective methods for treatment and prevention have not been established and further studies of MTSS are urgently required. An assessment of the static and dynamic alignment of the rearfoot in MTSS is necessary to understand the kinematics related to MTSS.

Static malalignment of the lower extremity has been shown to increase the risk of developing MTSS in athletes [12, 14]. In addition, many studies have reported the kinematic characteristics of MTSS, such as rearfoot eversion [15, 16], increased free moment [17] and loading rates [18], tibial shock [19], and hip external rotation [14, 20]. However these studies performed motion analysis with skin markers. Skin markers mounted upon externally identifiable bony landmarks of the foot do not follow the underlying individual skeletal segments during movement [21]. Motion analysis using skin markers does not allow the investigation of the subtalar joint (especially talus) movement [22]. Intracortical pins have also been used to more accurately measure in vivo rearfoot motion kinematics [23, 24], but this method is invasive and the restricted motion of bone pin markers makes these internal markers difficult to implement. In contrast, 3-dimensional to 2-dimensional (3D-2D) model registration techniques have been used for the analysis of rearfoot motion without the use of invasive markers [25, 26]. However, no prior study has used 3D-2D model registration to assess static and dynamic rearfoot behaviour in MTSS. The 3D-2D model allows to study the characteristics of patients with MTSS in terms of malalignment and kinematics, contributing to an improved understanding of MTSS.

Therefore, the aim of the present study was to investigate MTSS using 3D-2D model registration to obtain accurate structures of the rearfoot during the forward step. Hypothesis was set that MTSS patients would have greater subtalar joint pronation (dorsi flexion, eversion, external rotation), compared to healthy individuals, during the forward step.

## Methods

### Participants

The Ethics Committee of our institution approved the study protocol. A thorough explanation was provided to participants and consent was obtained from all participants. All participants belonged to the same university soccer team. The study population included eight male soccer players [age, 21.1 ± 2.1 y; body height, 174.6 ± 6.7 cm; body mass, 73.0 ± 5.7 kg; mean ± standard deviation (SD)] with MTSS and eight healthy soccer players [age, 19.6 ± 2.8 years; body height, 172.9 ± 5.3 cm; body mass, 71.0 ± 12.1 kg; mean ± standard deviation (SD)]. Between May 2011 and April 2013, Patients who had been diagnosed with MTSS by an experienced orthopaedic surgeon within a period of 6 months were recruited. The right lower leg was involved in six patients and the left lower leg was involved in two patients. MTSS was defined as exercise-induced pain in the posteromedial aspect of the tibia, and pain on palpation in an area of ≥ 5 cm in the posteromedial tibial region, based on the diagnostic criteria described by Yates and White [6]. All patients had experienced symptoms for at least two weeks, and MTSS patients who had undergone previous surgery were excluded. None of the healthy participants (eight right feet) had a history of surgery in their lower legs.

### Cineradiography (2D)

**Fig. 1:**
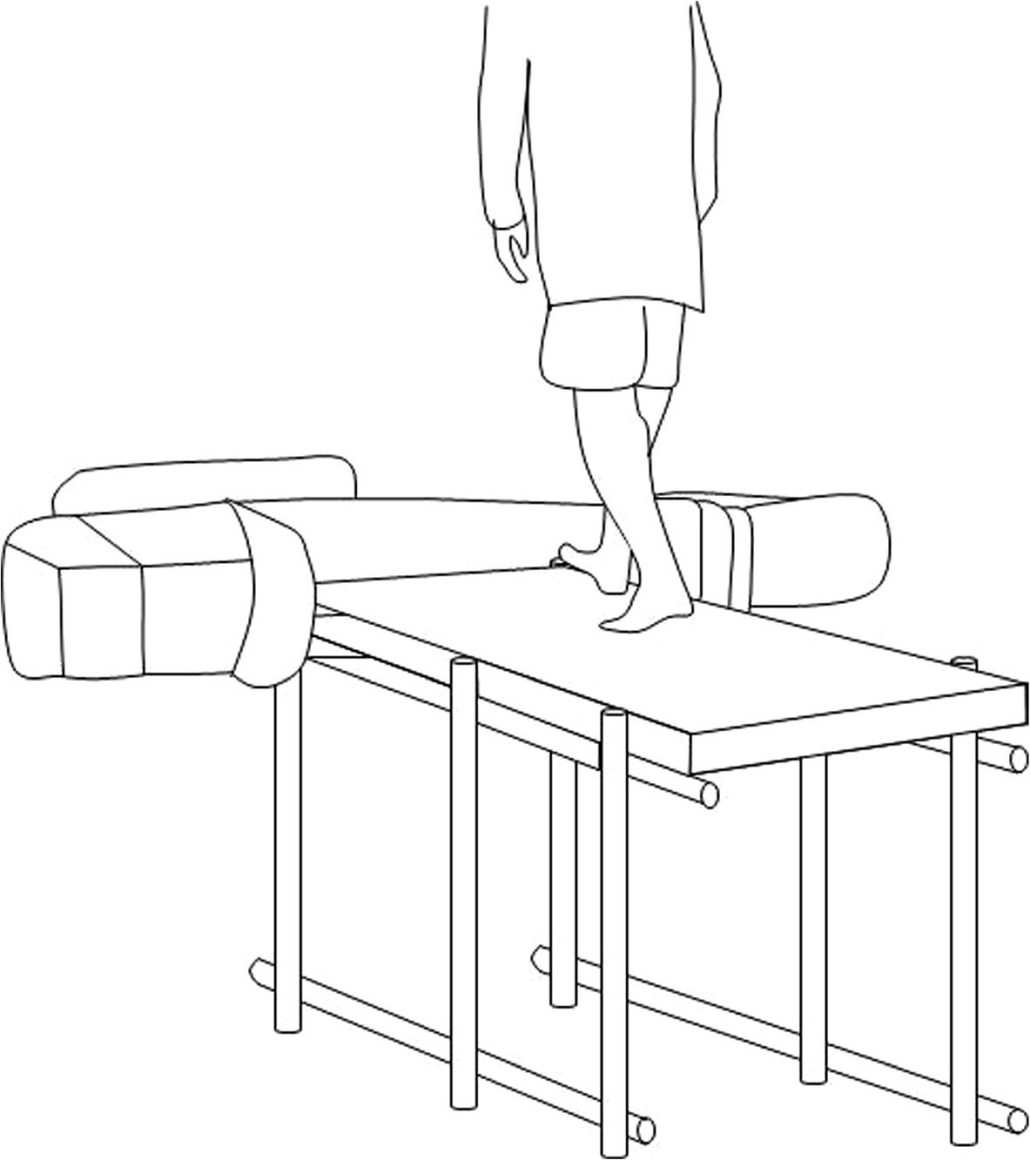
Setup of the data measurements. Participants performed a forward step on a cineragiography table (1 m × 1.5 m × 0.8 m, height-width-depth)

To investigate rearfoot movement during the stance phase of the forward step, we recorded forward step trials on a platform (Fig. 1). Participants first stood straight with both feet together to record the static lower extremity position (relaxed neutral standing position with knees straight). The participants then ran with a normal stride, with the second toe of the foot and heel contacting a line parallel to the direction of movement. We confirmed that the foot was parallel to the tape (parallel to direction of the movement) on the platform during all data collection. During the participants first step, we recorded the foot (right foot of healthy participants, foot corresponding to the side of MTSS) that was grounded (i.e., when one foot contacted the platform, the other foot was off the platform). The forward step pitch was set at 2.5 steps/s using a metronome. Each forward step trial was recorded using cineradiography (Infinix Celeve-l INFX-8000C, Toshiba Medical Inc., Tochigi-ken, Japan). Acquired orthogonal images squared the foot and the central ray. Eight inch square images were obtained at 60 frames/s, with an expected radiation exposure equivalent of 200 mA (1 ms) with an intensity of 50 kV, expected 15 mGy.

### Bone model (3D)

Geometric bone models of the tibia and talus/calcaneus were created from the study leg of all participants by computed tomography scans (IDT 16, Philips Healthcare, Best, The Netherlands) at a 0.4 mm slice thickness, scanning 15 cm above the rearfoot and below the calcaneus. The bone model ranges were defined using Image J (open source, http://imagej.nih.gov/ij/). Before segmenting the bone models, we converted dicom files to analysis files. Each subject’s bone image was loaded using ITK-SNAP (open source, http://www.itksnap.org/pmwiki/pmwiki.php). Each bone was segmented with cortical bone as the border, and these points (approximately 3000–10,000 points) were converted into polygonal surface models [3D bone models (tibia and talus/calcaneus)].

### Anatomical coordinate systems

**Fig. 2:**
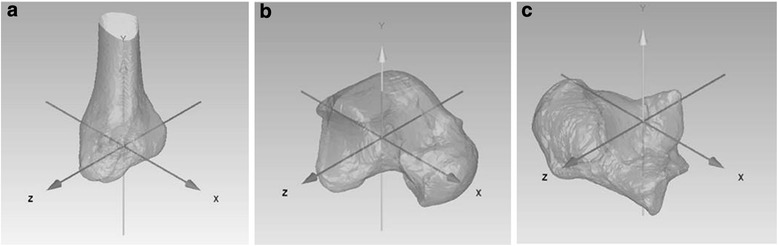
An example of the geometric bone model. (**a**: tibia, **b**: talus, **c**: calcaneus) After geometric bone models of the tibia and talus/calcaneus were created from computed tomography scans, 3D bone model anatomical coordinate systems were set

3D bone model anatomical coordinate systems were set (Geomagic Studio, 3D Systems, Rock Hill, SC, USA) following a combination of previously reported approaches (Fig. 2a, b, c) [27]. The axis of the tibia was defined as follows. The origin of the tibia was the flat center of the tibial plafond. The anteroposterior axis was defined as an orthogonal line to the anterior edge line of the tibial plafond passing through the origin. The superoinferior axis was defined as a line connecting the medial-lateral and anteroposterior center points of the distal tibial shaft at 5 and 10 cm above the joint surface, passing through the origin. The *Z*-axis was defined as a line perpendicular to the *X*- and *Y*-axes (Fig. 2a). The axis of the talus was defined as follows. A circle on the sagittal plane fit to two midpoints (the midpoint of the anteromedial and anterolateral edges and the midpoint of posteromedial and posterolateral edges of the trochlea tali) was defined. The origin of the talus was defined as the centre of the circle. The *Z*-axis was defined as a perpendicular line to the circle, passing through the origin. The *Y*-axis was defined as a line passing through the origin and circle at the highest point of the tibial tali. The *X*-axis was the cross product of the *Y* and *Z*-axes (Fig. 2b). The axis of the calcaneus was defined as follows. The origin was the center of a line connecting the most lateral point of the middle talar articular surface and posterior talar articular surface. The *X*-axis was a line parallel to the inferior calcaneus, passing through the origin. The *Y*-axis was a line parallel to the lateral wall of the calcaneus and perpendicular to the *X*-axis, passing through the origin. The *Z*-axis was the cross product of the *X*- and *Y*-axes (Fig. 2c). The angle of the rearfoot relative to the tibia, and the tibia relative to the laboratory coordinate system, were calculated according to Grood and Suntay [28].

### 3D to 2D registration technique

Three-dimensional kinematics of the tibia, talus, and calcaneus models were determined using a shape matching technique [29] and custom software (Joint Track, open source, http://sourceforge.net/projects/jointtrack/). A previous study that used an identical analysis indicated that the average intraobserver differences for the rearfoot were 0.35 mm for an out-of-plane translation and 0.85° for rotations [27]. The average interobserver differences for the rearfoot were 0.35 mm for an out-of-plane translation and 0.76° for rotations [27]. In this study, Intraclass correlation (ICC) was measured by comparing 3 measurements by the same investigator for each of the 16 ankles. The ICC were as follows: dorsi–plantar flexion, 0.92; inversion–eversion, 0. 81; internal–external rotation, 0.79.

**Fig. 3:**
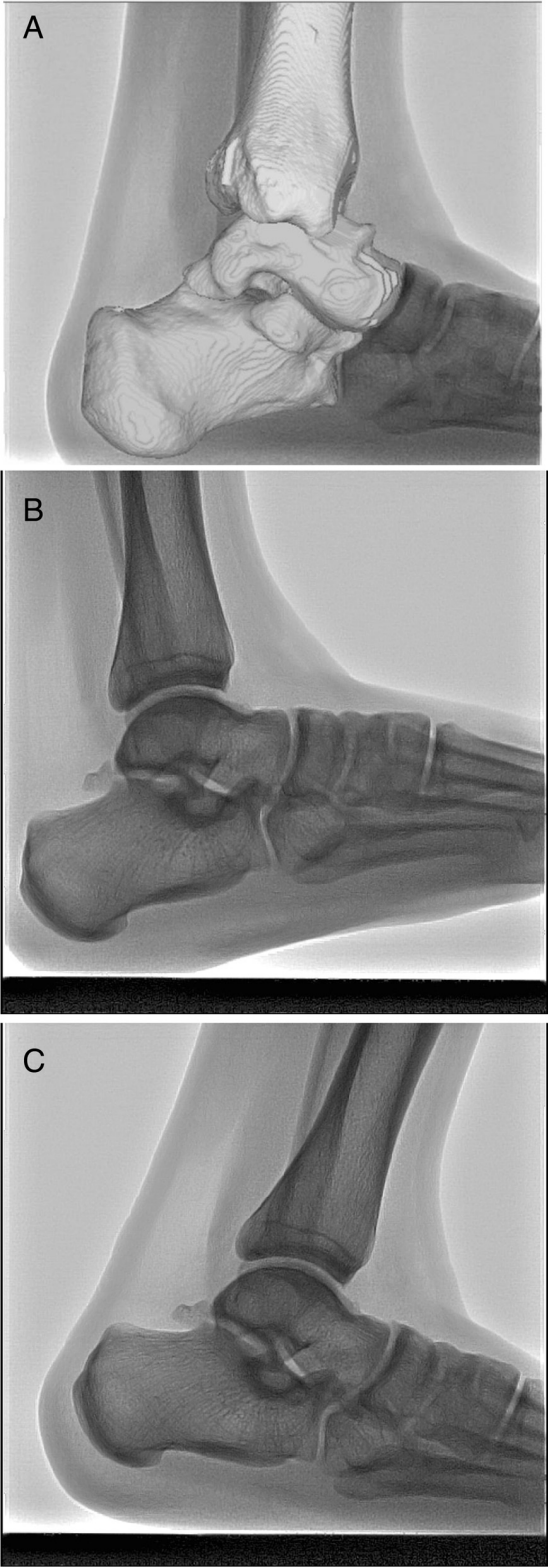
Cineradiography of left foot. **a**: The tibia, talus, and calcaneus models were matched, **b**: heel contact, **c**: heel off

The tibia, talus, and calcaneus models were matched with 2D images after modelling. In vivo rearfoot positions were then reproduced from the 3D rearfoot model (Fig. 3a). The following types of rearfoot motion were examined: motion of the talocrural joint (relative motion of the talus with respect to the tibia) and motion of the subtalar joint (relative motion of the calcaneus with respective to the talus). The rearfoot positions at different time intervals were then reproduced from a series of 3D rearfoot models from heel contact (Fig. 3b) to heel off (Fig. 3c) during a forward step. Plantarflexion/dorsiflexion was defined as the rotation along the mediolateral axis, internal/external rotation was the rotation along the superoinferior axis, and inversion/eversion was the rotation along the anteroposterior axis. All kinematics data were normalized relative to the weight-bearing stance phase where the heel strike occurred at zero percent and heel off at 100 % of the stance phase.

### Navicular height change and calcaneal pitch (Static alignment)

Before the forward step trials, the navicular height change was radiologically assessed. The perpendicular distance between the navicular tuberosity (mid point of navicular in a vertical direction) and a line connecting the lower end of the calcaneus and the lower end of the first metatarsal was then measured. Participants kept the non-weight bearing leg in front of the other leg (non-weight bearing). The test was repeated with the participants standing on both feet, shoulder width apart (weight-bearing). The values of the two measurements were subtracted to obtain the navicular height change. The ICC when the same examiner measured 16 ankles 3 times was 0.89. The calcaneal pitch was defined as the angle formed by drawing a line along the inferior border of the calcaneus and a line drawn in the horizontal from a standing lateral radiograph (weight-bearing) [30, 31].

### Statistical analysis

For all kinematics of subtalar joint motion and talocrural joint motion from heel strike to heel off, a two-way analysis of variance was conducted as condition (control and MTSS) × time. Whether the movement of the subtalar joint between groups was different in various stages of foot contact was checked. With significant interactions, the main effect for each factor was examined. Multiple comparisons confirmed that there was a correlation between the main effect and the interaction. An unpaired *t*-test was used to compare the differences in range of motion (dorsi–plantar flexion, inversion–eversion, internal–external rotation) of the control and MTSS participants. Ninety-five percent confidence intervals were calculated. Statistical significance was at *P* < 0.05. Effect size of the unpaired *t*-test was calculated in | t_0_ | ($\sqrt{1/\mathrm{n}1 + 1/\mathrm{n}2}$). The Kolmogorov-Smirnov test was carried out to check whether the probability distribution of the two populations was different; all the kinematics were normally-distributed.

## Results

### Subtalar joint

**Fig. 4:**
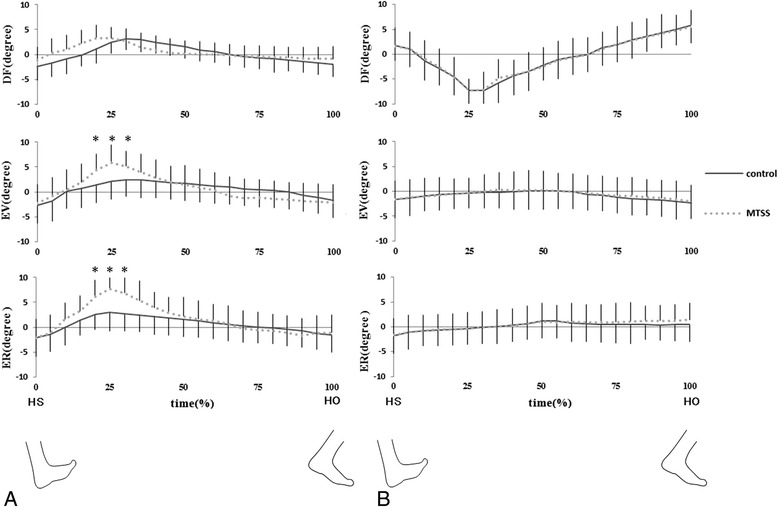
Mean kinematics data of **a**: subtalar joint motion and **b**: talocrural joint. All kinematics data from heel strike (HS) to heel off (HO). DF: dorsiflexion, EV: eversion, ER: external rotation. DF, EV, ER show positive values

There was a significant difference in the external rotation and eversion motions of the subtalar joint during 20–30 % of foot contact between MTSS patients and healthy participants. In the subtalar joint of MTSS patients, a significantly larger range of internal/external rotation and inversion/eversion motion was observed (*P* < 0.05) compared to healthy controls, from heel contact to heel off (Fig. 4). In contrast, no statistical difference was observed in subtalar plantarflexion/dorsiflexion between the two groups (Table 1).

**Table 1:** Range of motion, calcaneal pitch, and navicular height change in patients with and without MTSS. Joint range of motion during forward step in control participants and MTSS patients. Calcaneal pitch and navicular height change were measured as static alignment in control and MTSS patients

		Control (*n* = 8)	MTSS (*n* = 8)		
		MEAN ± SD	MEAN ± SD		ES
Subtalar joint	DF (+) / PF (−) (°)	6.1 ± 0.7	6.1 ± 1.2		0.1
	EV (+) / IV (−) (°)	5.3 ± 0.4	7.8 ± 1.3	*	2.6
	ER (+) / IR (−) (°)	5.5 ± 1.1	9.8 ± 0.9	*	4.2
Taloclural joint	DF (+) / PF (−) (°)	13.7 ± 2.2	13.6 ± 2.7		0.1
	EV (+) / IV (−) (°)	2.9 ± 1.0	3.4 ± 2.2		0.3
	ER (+) / IR (−) (°)	3.5 ± 1.0	3.7 ± 1.1		0.2
Calcaneal pitch	(°)	20.8 ± 5.2	14.3 ± 3.8	*	1.5
Navicular height change	(cm)	0.7 ± 0.2	0.9 ± 0.1	*	1.4
Time of Stance phase	(msec)	319 ± 9	323 ± 9		.0

*PF* Plantarflexion, *DF* Dorsiflexion, *IV* Inversion, *EV* Eversion, *IR* Internal rotation, *ER* External rotation, *ES* Effect size

*p < 0.05

### Talocrural joint

There was no significant difference in talocrural joint motion from heel strike to heel off between MTSS patients and healthy participants. There was also no significant difference in the range of all talocrural joint angles during the forward step between the MTSS patients and healthy participants (Table 1).

### Navicular height change and calcaneal pitch (Static alignment)

The navicular height change and calcaneal pitch in the MTSS patients were significantly lower than in healthy participants (Table 1; *P* < 0.05).

## Discussion

The purpose of this study was to reveal the characteristics of the static alignment and rearfoot motion during a forward step in MTSS patients. The subtalar joint of patients with MTSS was characterized by an increased range of motion in internal/external rotation and inversion/eversion during the forward step. In addition, the calcaneal pitch was lower and the navicular height change was greater in MTSS patients.

### Pronation in MTSS patients

In this study, MTSS patients showed more external rotation and eversion of the subtalar joint than that observed in normal participants. Moreover, the kinematics of the subtalar joint had a tendency in almost every case.

Campbell et al. reported an eversion peak of 8.7° for the normative rearfoot (calcaneus relative to tibia) during walking with biplane cineradiography [32]. With regard to the difference in the magnitude of eversion in this study compared to the Campbell study, this may have occurred because the rearfoot movement in this study included the subtalar joint and the talocrural joint.

The previous study is research related to the motion of dynamic foot in MTSS patients. Pohl et al., using logistic regression analysis, indicated that in female runners with tibial stress fractures, peak rearfoot eversion during a forward step was an important variable [15]. Standing foot pronation and longitudinal arch were greater in MTSS patients than in healthy participants [12, 33]. However, these previous studies employed uniplanar measurements. It is significant that our study used the 3D-2D model registration technique to evaluate the 3-dimensional kinematic characteristics of MTSS patients during a forward step.

Recently, several studies have investigated the relationship between MTSS and the kinematics of the lower extremity. The occurrence of tibial stress fractures in female runners was related to greater vertical loading rates of the lower extremity [17]. Athletes with shin splints had increased rear foot inversion and eversion during passive mobility [16]. Additionally, peak rearfoot eversion in athletes with MTSS [16] and peak rearfoot eversion of athletes with tibial stress fractures were significantly higher than those of normal participants [34]. Thus, the lower extremity that is affected by MTSS is believed to be associated with abnormal foot motion.

However, the pathophysiology of MTSS has been a subject of controversy.

The potential development of methods for preventing MTSS requires further study to investigate how subtalar pronation affects MTSS injury.

### Increased navicular height change in MTSS patients (Static alignment)

In this study, we found that static navicular height change was greater in MTSS patients compared to healthy participants. Several studies have examined the relationship between navicular drop and MTSS [10, 24, 35, 36]. The use of navicular drop to assess foot pronation has been especially useful in MTSS patients [24]. Additionally, a point-biserial correlation of 0.42 between the navicular drop test and the occurrence of MTSS was discovered, indicating a positive relationship between navicular drop, a measure of pronation, and MTSS injury [10]. In these studies, the navicular drop was greater in MTSS patients than in healthy participants [10, 24]. In contrast, Hubbard et al. reported that there was no relationship between MTSS and navicular drop [37]. These conflicting results are possibly caused by methodological differences. Almost all studies evaluated navicular drop using skin markers, and the reliability of this method has been reported as difficult to evaluate [34]. The present study used radiography, which is a more precise method, and we found that navicular height change was greater in MTSS patients.

### Calcaneal pitch in MTSS patients (Static alignment)

In the present study, the calcaneal pitch was lower in MTSS patients compared to healthy participants (14. 3° vs. 20.75°, *p* = 0.017), indicating that a low calcaneal pitch may be associated with MTSS. Other researchers found that the calcaneal pitch of normal participants was 20°–22.5° during standing [31, 38]. The calcaneal pitch angle is to patients with pes planus [30], and the presence of flat-feet types are used to patients with lower extremity injuries [39].

### Limitations

There are several limitations to the current study. First, considering the anatomy and functions of the rearfoot, more specific and precise studies concerning the subtalar joint are needed to validate the accuracy of the analysis. Second, our analysis range included only one forward step starting from a static standing position. Sports activities have a number of movements (e.g., running, cutting, stopping and turning, etc.), and therefore, further investigation of these motions in MTSS patients is needed. Third, the measurement of activity was limited to a single event to minimize radiation exposure. Importantly, the participants practiced the activity before the recorded event to ensure reproducibility and reduce inter-subject variability. Finally, the precision of the bone model in this study was not validated. Because this study performed matching from the sagittal plane of the cineradiograph, studies using biplane cineradiography with greater detail are necessary in the future. Further investigations will help more clearly elucidate these topics.

## Conclusions

The results of this study showed differences in the eversion and external rotation of the subtalar joint, navicular height change, and calcaneal pitch between normal participants and patients with MTSS. The subtalar of patients with MTSS is particularly associated with increased external rotation and eversion.

## Abbreviations

MTSS: Medial tibial stress syndrome
